# Pre-interventional assessment of right renal to right adrenal vein distance: Impact on procedure time and radiation dose in adrenal vein sampling

**DOI:** 10.1371/journal.pone.0279552

**Published:** 2022-12-30

**Authors:** Lennart Well, Clemens Spink, Alexander Lenz, Maxim Avanesov, Johannes Salamon, Gerhard Adam, Bjoern P. Schoennagel, Frank Oliver Henes, Peter Bannas

**Affiliations:** Department of Diagnostic and Interventional Radiology and Nuclear Medicine, University Medical Center Hamburg-Eppendorf, Hamburg, Germany; Medical University of Vienna, AUSTRIA

## Abstract

**Purpose:**

Adrenal vein sampling (AVS) is the reference standard for evaluation of lateralized hormone production in primary aldosteronism. We aimed to investigate the impact of pre-interventional right renal vein (RRV) to right adrenal vein (RAV) distance measurement on fluoroscopy time, contrast agent exposure and radiation dose during AVS.

**Materials and methods:**

Forty-five patients with primary aldosteronism undergoing AVS were enrolled in our retrospective study and divided into three groups. In the group *“ruler”* (n = 14), RRV-RAV-distances were determined pre-interventionally by cross-sectional imaging (CT/MRI) and AVS was performed by one interventional radiologist with limited experience in AVS. CT/MRI-derived and fluoroscopy-derived RRV-RAV-distances were correlated for aimed cannulation of the RAV. Patients in group *“no ruler”* (n = 24, three interventional radiologists with limited experience in AVS) and in group *“expert”*, (n = 7, one expert interventional radiologist) underwent AVS without pre-interventional estimation of RRV-RAV-distances. Procedure parameters (fluoroscopy time, contrast agent volume, radiation dose) of group *“ruler”* were compared to both other groups by Kruskal-Wallis rank-sum test.

**Results:**

Correlation of CT/MRI-derived and fluoroscopy-derived RRV-RAV-distances was good (r = 0.74;p = 0.003). The median RRV-RAV-distance was 4.5cm at CT/MRI (95%-CI:4.2–5.0cm) and 4.0cm at fluoroscopy (95%-CI:3.8–4.5cm). Fluoroscopy time (p<0.0001), contrast agent exposure (p = 0.0003) and radiation dose (air kerma and dose area product both p = 0.038) were significantly lower in group *“ruler”* compared to group *“no ruler”* (all p<0.05), and similar to group *“expert”* (all p>0.05).

**Conclusions:**

CT/MRI-derived pre-interventional renal-adrenal vein distance measurements correlate well with angiographic distance measurements. Pre-interventional estimation of the RRV-RAV-distance allows for aimed cannulation of the RAV with potential reduction of fluoroscopy time, contrast agent exposure and radiation-dose during AVS.

## Introduction

Primary aldosteronism is the most common cause of secondary hypertension [1]. Early diagnosis of primary aldosteronism provides the opportunity to prevent irreversible cardiovascular damage and end-stage renal disease [2]. The most common causes for primary aldosteronism are bilateral idiopathic adrenal hyperplasia and unilateral aldosterone producing adrenal adenomas [1, 3]. Patients with unilateral aldosterone producing adrenal adenomas can be cured with an adrenalectomy [4]. Therefore, it is of importance to correctly identify whether aldosterone excess is based on one or both adrenal glands. In cases of unilateral aldosterone excess, correct identification of the adrenal gland is vital for planning a unilateral adrenalectomy [2].

Adrenal vein sampling is the established reference standard for evaluation of lateralized hormone production [5–8]. Unfortunately, adrenal vein sampling is a difficult interventional procedure and particularly sampling of the right adrenal vein (RAV) can be demanding [3, 9, 10] with reported success rates ranging from 59% - 97% [9, 11]. The RAV is very small and drains directly into a long segment of the inferior vena cava, stretching between the renal veins and the hepatic veins, impeding expeditious, aimed detection and cannulation [9, 12–14]. Additionally, anatomical variations of the location of the right adrenal vein compared to the inferior vena cava, different angulations or a common trunk of the right adrenal vein with an accessory hepatic vein can further impede successful adrenal vein sampling [15]. The difficulty in detection and cannulation of the RAV results in long fluoroscopy times and high radiation exposure of patients undergoing adrenal vein sampling [11, 16].

Therefore, different techniques to improve adrenal vein sampling in general and sampling of the RAV in particular have been proposed [9, 14, 17–24]. These strategies can be divided into pre- and peri-interventional approaches. Pre-interventional approaches aim to improve delineation of adrenal veins by using improved cross-sectional imaging techniques, e.g. dual-energy CT for increased vascular contrast [17, 25]. Peri-interventional techniques include the use of Dyna-CT or C-arm CT for validation of correct catheter placement [18, 20, 26], use of cortisol testing kits for fast validation of correct cannulation of the adrenal veins [19], adjustment of catheter-shape to RAV-anatomy during adrenal vein sampling [24] or identification of specific landmarks which may aide cannulation of the RAV [27, 28].

However, an easily applicable combination of pre- and peri-interventional approaches has not been introduced yet. We hypothesized that pre-interventional estimation in CT and MRI of the right renal vein (RRV) to RAV-distance with peri-interventional correlation using a radiopaque ruler may facilitate and accelerate identification and cannulation of the RAV.

Therefore, the aim of our study was to investigate the impact of pre-interventional RRV to RAV-distance measurement on fluoroscopy time, contrast agent exposure and radiation dose during adrenal vein sampling (AVS).

## Materials and methods

The institutional review board approved this retrospective single-center study and waived the requirement for informed consent. All procedures were in accordance with the principles of the 1964 Declaration of Helsinki and its later amendments. All procedures being performed were part of the routine care. All clinical and imaging data were anonymized.

### Study population

Inclusion criteria for this study were: history of hypertension with clinically suspected primary aldosteronism and subsequent referral for adrenal vein sampling for identification of lateralized hormone production in our clinic between December 2014 and December 2019 (n = 55). Successful performance of AVS by either “non-expert” interventional radiologists (IR experience of ≤5 years and ≤10 AVS procedures) or an “expert” interventional radiologist (IR experience of ≥ 10 years and ≥50 AVS procedures). Successful AVS was defined as a sufficient step-up of cortisol level in the acquired samples as described below.

Exclusion criteria were: unsuccessful adrenal vein sampling (n = 8). Performance of adrenal vein sampling by interventional radiologists who were neither “non-expert” nor “expert” interventional radiologists (n = 2).

Thus, 45 patients with successful venous sampling of both adrenal glands were included in this study (27 male; mean age: 52.6 ± 9.2 years; mean BMI: 29.7 ± 12.7) for evaluation of fluoroscopy time, contrast agent exposure and radiation dose.

### Adrenal vein sampling protocol

Thirty minutes before intervention, stimulation of cortisol and aldosterone release by infusion of cosyntropin (Synacthen^®^, Novartis Pharmaceuticals, Dorval, Canada) (0.25 mg in 500 ml of 5% dextrose in water, infused at 100 ml/h) was initiated and continued throughout the procedure [29–31]. All AVS procedures were performed using single-plane digital subtraction angiography (AlluraClarity, Philips Healthcare, Best, The Netherlands). The number of frames per second during DSA was 2/s.

Venous access was established by percutaneous transfemoral vein catheterization using a 5-French (5F) vascular sheath. 5F catheters (Terumo Europe, Leuven, Belgium) with custom made side holes 2 mm distant from the tip were used for venous sampling of the inferior vena cava and adrenal veins: C2 Cobra catheters were used for venous sampling of the left adrenal vein while C2 Cobra or Sidewinder-2 catheters (Terumo Europe, Leuven, Belgium) were used for sampling of the right adrenal vein. Correct positioning of the catheter in the left or right adrenal vein was confirmed by careful venography with gentle injection of contrast medium by hand in resting end-expiratory position before sampling [9]. For sampling, one sample per sampling location (inferior vena cava, left adrenal vein, right adrenal vein) with a minimum of 5 ml blood was collected. Intraprocedural cortisol measurements were used during adrenal vein sampling to confirm correct cannulation of adrenal veins [19].

Sampling of the left adrenal vein was performed in all patients by advancing the 5F-catheter down the left renal vein for 3–4 cm over a guidewire. Subsequently, the tip of the catheter was turned cranially and the left adrenal vein was cannulated while retreating the catheter [9].

Sampling of the RAV was performed by probing the posterior wall of the IVC with the 5F C2 Cobra catheter at a level chosen by the interventional radiologist and was then extended in an arc of up to 90° to the right until the RAV was identified [9]. Venous sampling was considered successful with a selectivity index (cortisol concentration_adrenal vein_ / cortisol concentration_inferior vena cava_) of ≥ 3 [32]. No coaxial microcatheters were used during AVS.

All AVS procedures were performed by the respective interventional radiologists on call (n = 5). All interventional radiologists participating in this study had undergone interventional radiology fellowship training.

Patients were retrospectively allocated to three groups: i) *“ruler”*, ii) *“no ruler”*, and iii) “*expert”* depending on the experience of the interventional radiologist and the used technique to sample the right adrenal vein as described below.

### Group *“ruler”*

**Fig 1:**
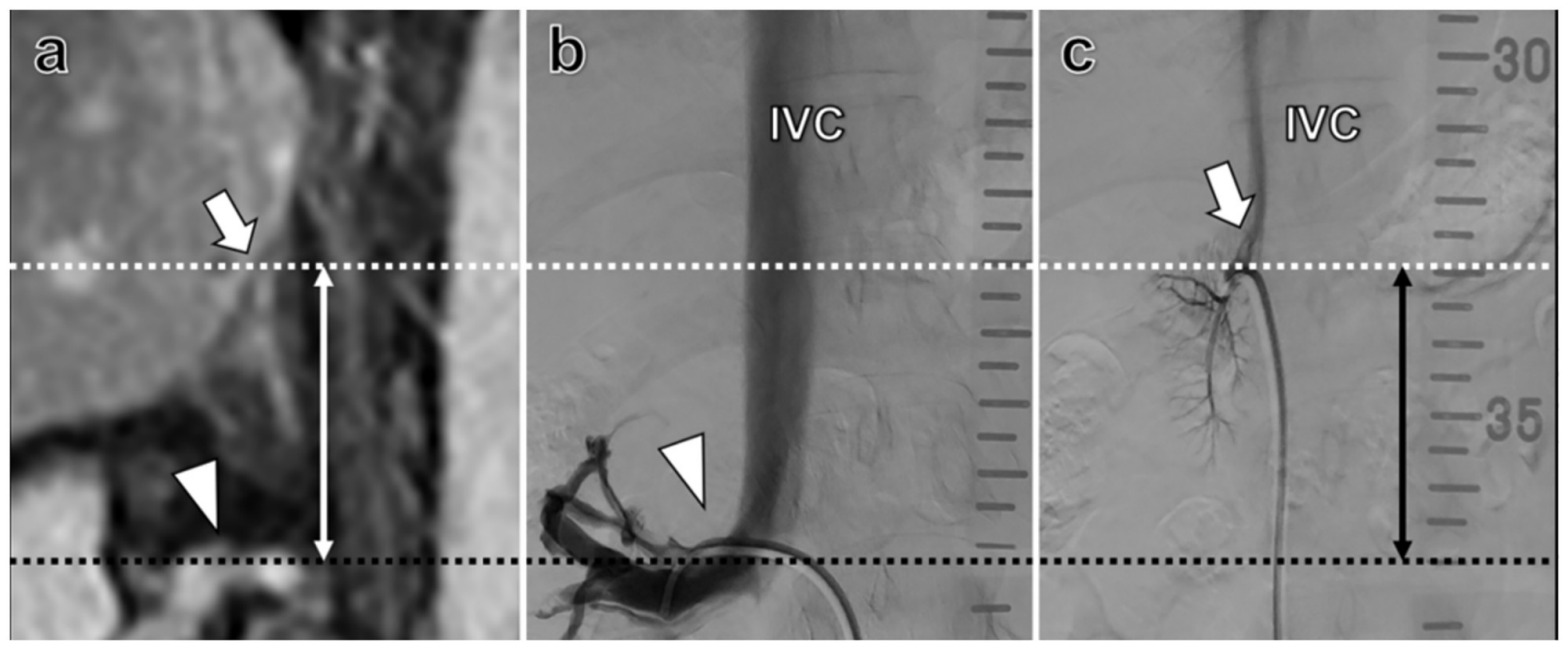
Estimation of pre- and peri-interventional right renal vein to right adrenal vein (RRV-RAV) distance in a 35-year-old woman with suspected Conn syndrome. **(a)** Para-coronal fat-saturated T1-weighted contrast-enhanced MRI illustrates pre-interventional estimation of RRV-RAV distance. **(b, c)** Illustration of angiographic RRV-RAV distance estimation. During adrenal vein sampling, a radiopaque planning ruler was placed underneath each patient and aligned along the spine. The right renal vein was then probed with a 5F-C2-catheter and delineated by contrast agent injection (arrowhead) in resting end-expiratory position. Aimed cannulation of the right adrenal vein was then focused at the height of the pre-interventionally determined RRV-RAV-distance and confirmed by contrast injection (arrow). Black dotted line marks the upper border of the renal vein. White dotted line marks the level of the right adrenal gland/RAV. IVC = Inferior vena cava.

In group *“ruler”*, AVS was performed by an interventional radiologist with limited experience in AVS (PB) (IR fellow with 4 years of experience in IR; previously performed AVS procedures n = 3). The group *“ruler”* consisted of 14 patients (8 male; age: 51.9 ± 11.5 years; BMI: 27.5 ± 5.2). All patients had received CT or MRI examinations prior to the procedure for evaluation of adrenal hyperplasia or adrenal adenoma. The interventional radiologist determined the RRV-RAV distance using the pre-interventional cross-sectional imaging studies (CT/MRI) prior to the interventional procedure using a standard Picture Archiving and Communication System (PACS) workstation allowing for distance measurements (Fig 1a). Delineation of the right adrenal vein by cross-sectional imaging was feasible in 12/14 patients (85.7%). In two cases (14.3%), in which delineation of the right adrenal vein was not feasible, the hilum of the right adrenal gland was used as a landmark to determine RRV-RAV distance [9].

During adrenal vein sampling, a radiopaque planning ruler was placed underneath each patient and aligned along the left side of his spine. The right renal vein (RRV) was probed with a 5F-catheter and delineated by gentle contrast agent injection by hand (Fig 1b). Aimed cannulation of the RAV was then focused on the height of the pre-interventionally determined RRV-RAV-distance and confirmed by contrast injection (Fig 1c).

### Groups without ruler: *“no ruler” and “expert”*

Patients in group *“no ruler”* were examined by three other interventional radiologists with limited experience in AVS (JS, BPS, FOH) (IR fellows with a mean experience of 4 years in IR (range 3–5 years); previously performed AVS procedures (mean n = 5.3; range: 3–9). Due to the limited experience of the non-expert interventional radiologists, AVS was performed when an experienced interventional radiologist was on-site and would have been able to aid during the procedure.

These radiologists did not perform RRV-RAV-measurements before or during adrenal vein sampling. The group *“no ruler”* consisted of 24 patients (15 male; age: 51.5 ± 7.8 years; BMI: 31.4 ± 17.0).

Group *“expert”* consisted of 7 patients (5 male; mean age: 58.0 ± 7.6 years; mean BMI: 28.5 ± 6.3) who were examined by one expert interventional radiologist with 12 years of experience (GA) in adrenal vein sampling without RRV-RAV-measurements before or during adrenal vein sampling.

The interventional radiologists who did not use a ruler during AVS (groups *“no ruler”* and *“expert”*) routinely assessed only the anatomy and location of both adrenal glands on cross-sectional imaging before the procedure.

There were no significant differences regarding age or BMI between the groups “*ruler”*, “*no ruler”*, and *"expert”* (all p>0.05).

### Procedural parameters and documentation

Pre-interventional CT/MRI-derived RRV-RAV distances and venography-derived RRV-RAV-distances were documented for group *“ruler”*.

Radiation Dose Structured Reports (RDSR) were automatically created by the computer workstation of the angiographic system after each procedure and transferred into the PACS. RDSR contain fluoroscopy time (FT), cumulative air kerma (AK) and dose area product (DAP). The total amount of contrast agent (Imeron 300; Bracco Imaging Deutschland GmbH, Konstanz; Germany) was recorded for each procedure in the documented material usage. These procedural parameters were documented for all patients.

### Statistical analysis

Normally distributed continuous data are presented as mean ± standard deviation, not normally distributed continuous data are given as median with interquartile range (IQR). Normal distribution of continuous data was evaluated by the Shapiro-Wilk test.

Comparison of procedure parameters was performed using the Kruskal-Wallis rank sum test with post-hoc analysis by Dunn’s multiple comparison test. Pairwise comparison was performed using Wilcoxon signed rank test. All tests were two-sided.

Measurements of RRV-RAV distances as determined by cross-sectional imaging or fluoroscopy were correlated by Spearman correlation analysis. P-values <0.05 were considered statistically significant. Statistical analysis was performed using GraphPad Prism 5.0 for Windows (GraphPad Software Inc., La Jolla, CA, USA).

## Results

### Estimation of RRV-RAV distance

Right renal vein to right adrenal vein (RRV-RAV) distances were derived from pre-interventional cross-sectional imaging (CT/MRI) in 12/14 patients (85.7%) and from peri-interventional fluoroscopy in all 14 patients (100%) of group “*ruler”*.

**Fig 2:**
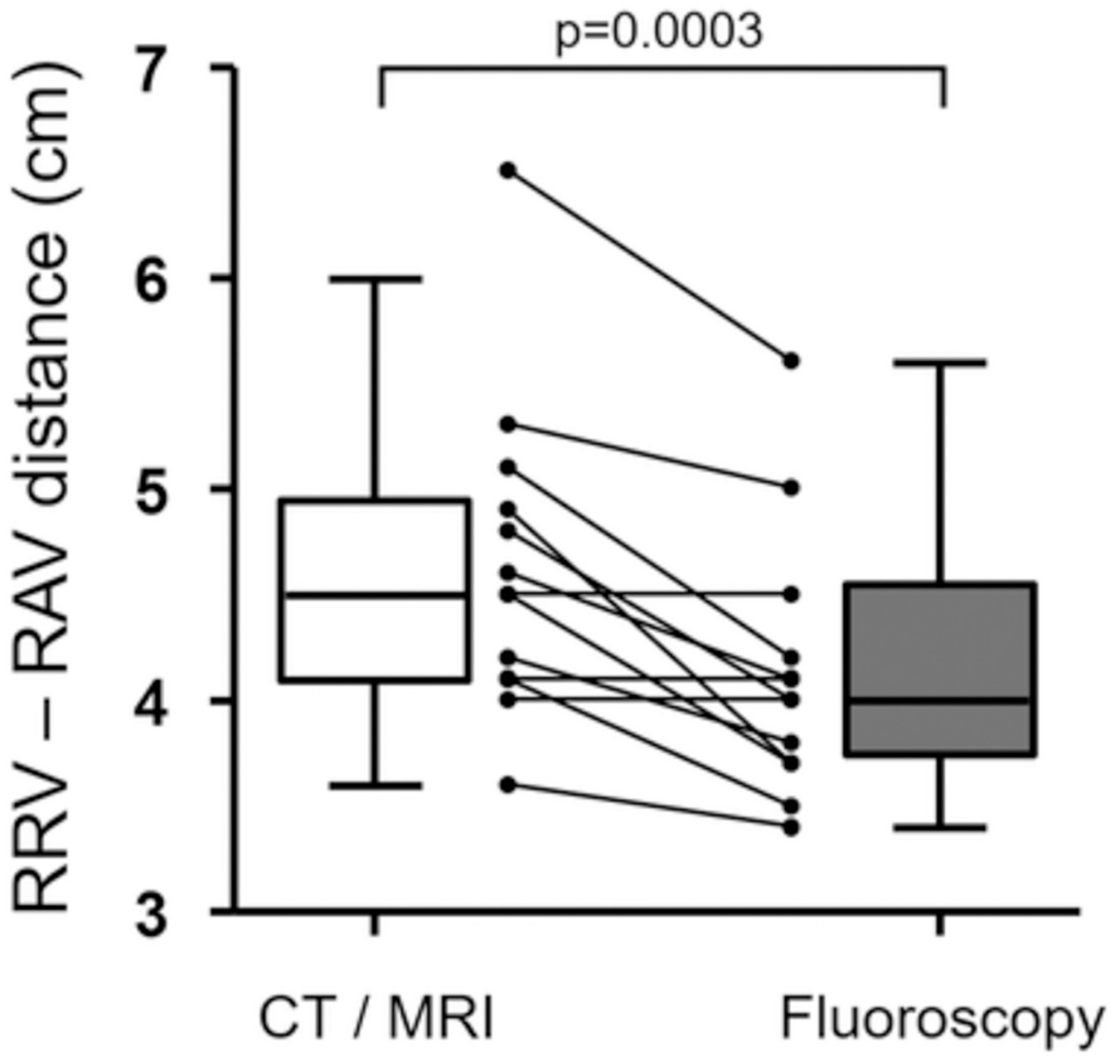
Comparison of right renal vein (RRV) to right adrenal vein (RAV) distance between pre-interventional cross-sectional imaging (CT/MRI) and peri-interventional fluoroscopy. Line graphs allow for direct comparison of individual RRV-RAV distances for each patient derived from both CT/MRI and fluoroscopy. Pre-interventional CT/MRI-derived RRV-RAV distances revealed a good correlation with peri-interventional fluoroscopy-derived RRV-RAV distances (r = 0.74; p = 0.003). The median (RRV-RAV) distance derived from cross-sectional imaging (CT/MRI) was 4.5 cm (95%-CI: 4.2–5.0 cm), while the median RRV-RAV distance derived from fluoroscopy was 4.0 cm (95%-CI: 3.8–4.5 cm) (mean difference: 0.4 ± 0.3 cm; p = 0.0003). Boxes range from first to third quartile, horizontal lines indicate the median, and whiskers indicate 5^th^-95^th^ percentiles.

Pre-interventional CT/MRI-derived RRV-RAV distances correlated with peri-interventional fluoroscopy-derived RRV-RAV distances (r = 0.74;p = 0.003) (Fig 2). The median RRV-RAV distance derived from cross-sectional imaging (CT/MRI) was 4.5cm (IQR: 4.2–5.0 cm), while the median RRV-RAV distance derived from fluoroscopy using the radiopaque ruler was 4.0cm (IQR: 3.8–4.5 cm) (mean difference: 0.4 cm ± 0.3 cm; p = 0.0003).

### Adrenal vein sampling procedures with and without ruler

During adrenal vein sampling, no major or minor complications were recorded.

**Fig 3:**
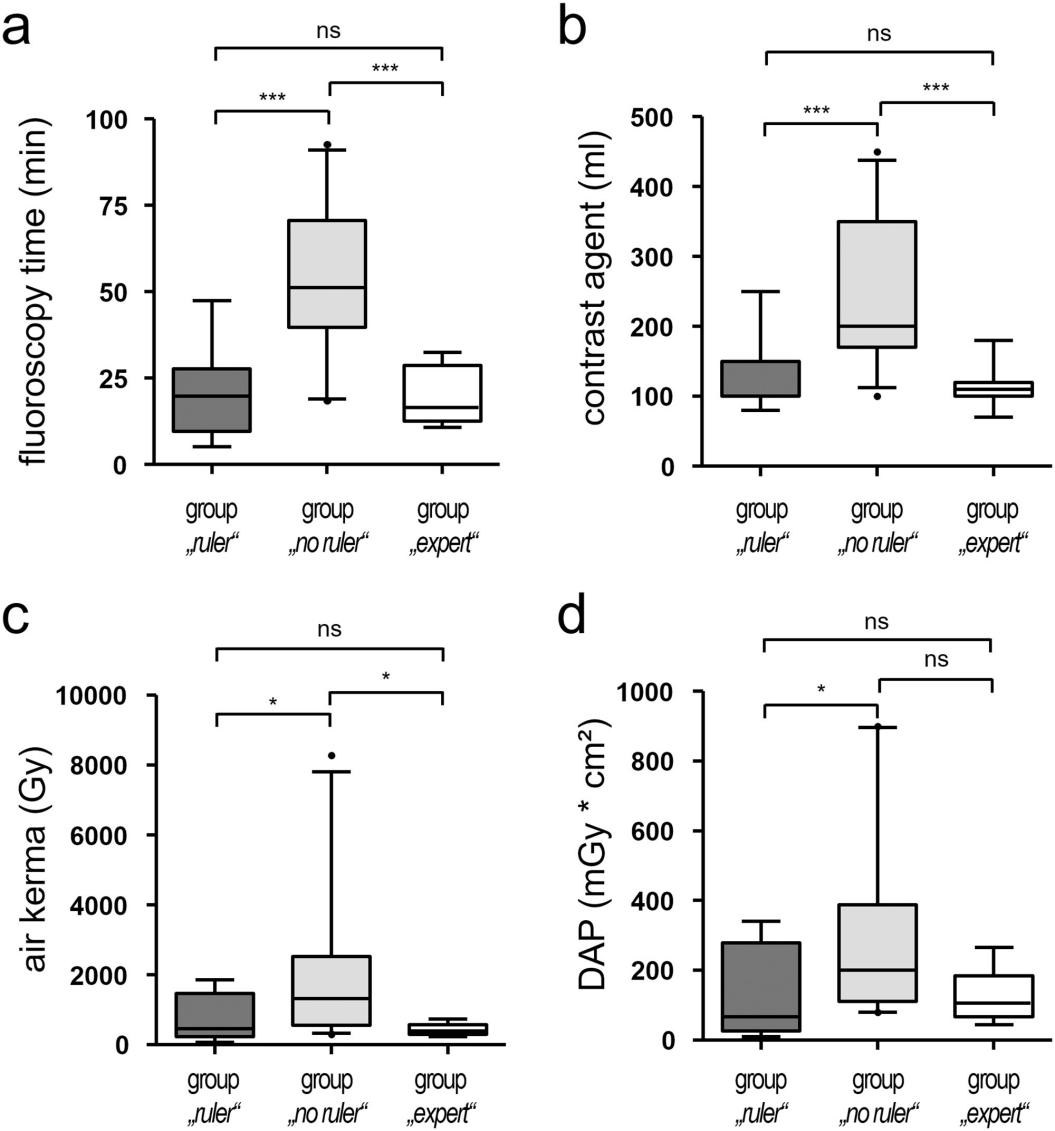
Comparison of fluoroscopy time (a), contrast volume (b), air kerma (AK) (c), and dose area product (DAP) (d) during adrenal vein sampling. Procedures were performed either by one non-expert radiologist using a radiopaque ruler (group *“ruler”*), by three non-expert radiologists without ruler (group *“no ruler”*), or by one expert radiologist without ruler (group *“expert”*) to determine the distance of right renal vein (RRV) to right adrenal vein (RAV). Box plot analyses illustrate significantly lower fluoroscopy time **(a)**, volume of contrast agent **(b)**, AK **(c)**, and DAP **(d)** in group *“ruler”* compared to group *“no ruler”*, without being statistically significantly different from group *“expert”*. Boxes range from first to third quartile, horizontal lines indicate median, and whiskers indicate 5^th^-95^th^ percentiles. Ns: not significant, *: p<0.05, ***: p<0.001.

Fluoroscopy time during adrenal vein sampling procedures was 20.4 ± 11.9 min in group *“ruler”* and significantly lower than in group *“no ruler”* [54.3 ± 20.1 min; p<0.0001], but not statistically significant different compared to group *“expert”* [19.4 ± 8.3 min; p = 0.97] (Fig 3a).

Volume of injected contrast agent was 138.6 ± 46.1 ml in group “*ruler*” and significantly lower than in group *“no ruler”* [251.3 ± 97.9 ml; p = 0.0003], but without a statistically significant difference when compared to group *“expert”* [114.3 ± 33.6 ml; p = 0.30] (Fig 3b).

Air kerma (AK) was 456 mGy [IQR: 230–1460] in group *“ruler”* and significantly lower than in group *“no ruler”* [1327 mGy; IQR: 558–2523; p = 0.038], but without a statistically significant difference when compared to group *“expert”* [398 mGy; IQR: 306–573; p = 0.85] (Fig 3c).

The lowest dose area product (DAP) was observed in group *“ruler”* [67 Gy*cm^2^; IQR: 26–278] and was significantly lower than in group *“no ruler”* [DAP: 201 Gy*cm^2^; IQR 111–388; p = 0.038], but without a statistically significant difference when compared to group *“expert”* [105 Gy*cm^2^; IQR: 67–185; p = 0.85] Fig 3d). All data on procedural parameters are provided in S1 Table.

## Discussion

Our study reveals that pre-interventional estimation of RRV-RAV-distances with peri-interventional correlation allows for aimed cannulation of the right adrenal vein, resulting in reduction of fluoroscopy time, contrast agent exposure and radiation-dose during adrenal vein sampling, when compared to adrenal vein sampling performed by radiologists with minimal experience and without estimation of RRV-RAV-distance.

CT/MRI-derived RRV-RAV-distances demonstrated good correlation with fluoroscopy-derived RRV-RAV-distances. However, the median CT/MRI-derived RRV-RAV-distance (4.5 cm) was higher than the median fluoroscopy-derived RRV-RAV-distance (4.0 cm).

Knowing this offset of 0.5 cm is of clinical importance, as it will allow for even more precise cannulation during future procedures when applying our proposed technique. Also, the estimated interquartile range of 3.7–4.4 cm for fluoroscopy-derived RRV-RAV-distances has a practical clinical value: The narrow range indicates that aimed cannulation during adrenal vein sampling within these limits will aid expeditious detection and cannulation of the right adrenal vein even without pre-interventional estimation of the RRV-RAV-distance. A potential reason for the difference in RRV-RAV distances between fluoroscopy and CT/MRI might be caused by different breath-hold positions. However, all venograms during AVS in this study were performed in the resting end-expiratory position which is similar to the breath-hold position during CT/MRI.

In our study, pre-interventional estimation of RRV-RAV-distances with peri-interventional correlation resulted in procedure parameters (FT, AK, DAP, contrast volume) that were significantly lower than those in a group of patients treated by radiologists with similar experience and comparable to a group of patients treated by an experienced interventional radiologist. These observations underline the usefulness of the proposed method, especially for non-expert interventional radiologists when learning the technically demanding procedure of adrenal vein sampling. Future studies are warranted to determine if experienced interventional radiologists also benefit from our proposed planning ruler approach.

Different technical approaches for improvement of adrenal vein sampling have been proposed. These included improvement of pre-interventional imaging, such as the use of thin-slice CT, dual adrenal venous phase CT or dual-energy CT [14, 17, 33]. Others compared pre-interventional CT and MRI and sought to identify anatomic landmarks such as accessory hepatic veins or vertebrae to facilitate cannulation of the RAV during AVS [27, 28]. Some authors focused on improvement of peri-procedural methods, such as rapid cortisol measurements [19, 20], evaluation of differently shaped catheters [24] or use of cone beam CT during AVS [18, 34]. Only few approaches for improvement of adrenal vein sampling combine pre- and peri-interventional methods such as the simple to use planning ruler approach successfully evaluated in our study. Technically more demanding approaches have been described by Morita et al or Busser et al who propose co-registration of pre-interventionally acquired CTs and real-time fluoroscopy during adrenal vein sampling [21, 35]. In the study by Morita et al, the proposed co-registration technique significantly reduced fluoroscopy time (95.6 vs. 108.4 min), DAP (43.1 vs 72.2 Gy*cm^2^) and contrast volume (54.6 vs 65.7 ml) [35].

There are several limitations to our study. An intraindividual comparison, i.e. adrenal vein sampling performed with or without a planning ruler by the same radiologist has not been performed due to the retrospective nature of our study. For the same reason, the specific times required for successful cannulation of the RAV were not recorded. Furthermore, only one radiologist performed AVS in group *“ruler”* with a planning ruler, while three other radiologists performed AVS in group *“no ruler”* and one additional radiologist performed AVS in group *“expert”*. Thus, different operators were using different methods. However, the experience in AVS was comparable between the radiologist who performed AVS in group “*ruler*” (4 years) and the group of radiologists who performed AVS in group “*no ruler*” (4 years, range 3–5). A potential bias due to different technical prowess between the radiologists responsible for group “*ruler*” and group *“no ruler”* cannot be excluded. Another important limitation is the small number of patients included in our study and within each of the subgroups. We are aware that our results might be overoptimistic and that future randomized studies are needed to confirm the results of our retrospective study.

## Conclusions

In conclusion, the results of our study demonstrate that CT/MRI-derived pre-interventional renal-adrenal vein distance measurements correlate well with angiographic distance measurements. Pre-interventional estimation of the RRV-RAV-distance allows for aimed cannulation of the RAV with potential reduction of fluoroscopy time, contrast agent exposure and radiation-dose during AVS. Our proposed planning ruler approach may be of particular use for interventional radiologists with limited experience in adrenal vein sampling and is intended to help improving the performance of this challenging procedure.

## Supporting information

S1 Table (XLSX)
